# Approach to Cataract Surgery in an Ebola Virus Disease Survivor with Prior Ocular Viral Persistence

**DOI:** 10.3201/eid2607.191559

**Published:** 2020-07

**Authors:** Jill R. Wells, Ian Crozier, Colleen S. Kraft, Mary Elizabeth Sexton, Charles E. Hill, Bruce S. Ribner, Sina Bavari, Gustavo Palacios, William A. Pearce, Russell Van Gelder, Hans Grossniklaus, Lisa Cazares, Xiankung Zeng, Jessica G. Shantha, Steven Yeh

**Affiliations:** Emory University School of Medicine, Atlanta, Georgia, USA (J.R. Wells, W.A. Pearce, H. Grossniklaus, J.G. Shantha, S. Yeh);; National Cancer Institute, Frederick, Maryland, USA (I. Crozier);; Emory University Hospital, Atlanta (C.S. Kraft, M.E. Sexton, C.E. Hill, B.S. Ribner);; United States Army Medical Research Institute of Infectious Disease, Frederick (S. Bavari, G. Palacios, L. Cazares, X. Zeng);; University of Washington, Seattle, Washington, USA (R. Van Gelder)

**Keywords:** Cataracts, cataract surgery, Ebola disease survivor, Ebola virus, ocular biopsy, panuveitis, phacoemulsification surgery, sexually transmitted infections, viruses, visual impairment, Ebola virus disease, EVD

## Abstract

A 46-year-old patient with previously documented Ebola virus persistence in his ocular fluid, associated with severe panuveitis, developed a visually significant cataract. A multidisciplinary approach was taken to prevent and control infection. Ebola virus persistence was assessed before and during the operation to provide safe, vision-restorative phacoemulsification surgery.

The 2013–2016 Ebola virus disease (EVD) outbreak in western Africa was unprecedented in magnitude, leading to the largest cohort of EVD survivors in history (*1*). Thousands of survivors were at risk for ophthalmic, mental health, and other EVD-associated health conditions, as well as for Ebola virus (EBOV) persistence in immune-privileged organs, including the eyes, central nervous system, and reproductive organs (*1*–*6*). Three outbreaks have also occurred in the Democratic Republic of Congo from 2016 through 2020 (*7*,*8*), underscoring the potential public health impact of medical care for EVD survivors. 

Elsewhere we reported the finding of EBOV persistence in the aqueous humor, associated with sight-threatening panuveitis in a healthcare worker who is an EVD survivor (*2*). Subsequent follow-up visits showed multiple recurrences of anterior and intermediate uveitis, which required treatment with topical corticosteroids (*9*). The patient subsequently developed a visually significant cataract. Because of uncertainty about whether EBOV could persist in the eye, doctors had to consider this when developing an approach to treatment. The resulting treatment plan included revised workflow for phacoemulsification, including laboratory specimen analysis. 

## Studies

The patient, a 46-year-old healthcare worker from the United States with a history of acute, severe panuveitis associated with iris heterochromia (Figure, panel A), hypotony, and persistence of ocular EBOV, had been treated with oral corticosteroids, favipiravir, periocular triamcinolone acetonide (40 mg/mL), and intensive topical difluprednate for 2 years prior to seeking treatment for the cataract (*2*,*9*). During 2015–2016, the patient experienced 2 episodes of recurrent anterior uveitis. During these episodes, we used reverse transcription PCR (RT-PCR) to test for EBOV RNA in the aqueous humor; results were negative. 

Over the following 3 months, the patient experienced progressive vision loss without pain. On follow-up examination, the iris heterochromia had resolved. Visual acuities were 20/20 in the right eye and 20/125 in the left eye. Slit lamp examination results were unremarkable for the right eye but showed a 4+ posterior subcapsular cataract in the left eye. While the patient awaited surgical treatment, recurrent anterior uveitis prompted doctors to prescribe a tapering course of topical corticosteroids. Results from RT-PCR for EBOV RNA of an aqueous humor aspirate were negative. We documented 3 months of disease inactivity prior to cataract surgery. During this time, his visual acuity in the left eye declined to the hand motions level, and he developed an intumescent uveitic cataract (Figure, panels B and C). 

**Figure:**
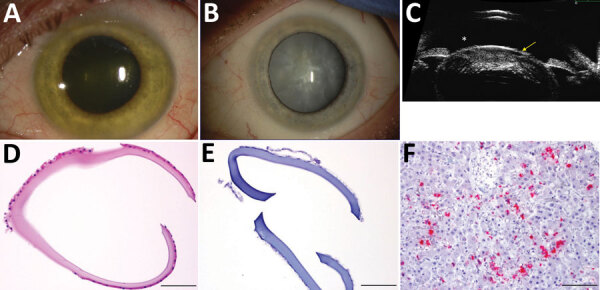
Cataract surgery in an Ebola virus disease survivor with prior ocular viral persistence. A) Slit lamp image shows green iris hue when patient developed panuveitis with heterochromia. B) The greenish coloration resolved but a dense intumescent cataract developed as shown in the second slit lamp image. C) Ultrasound biomicroscopic examination demonstrates the bulging of the anterior lens capsule (yellow arrow) and shallowing of the anterior chamber (*), which presents an increased risk for anterior and posterior capsular tears during surgery. D) Hematoxylin and eosin staining shows thickening of the removed anterior capsule. E) Results were negative for Ebola virus RNA by in situ hybridization. F) Positive control tissue.

After evaluating the risk of exposure during the operation, the surgical team chose to use personal protective equipment (PPE) including a surgical hat, mask, and fluid-impervious scrub booties. Additional PPE was not used because they considered the amount of fluid in the surgical field too small to present a significant splash risk (Appendix). The surgery was scheduled as the final procedure in the day, recognizing the possibility of the ocular fluids testing positive for EBOV RNA. In this way, the room could be terminally cleaned after surgery without disrupting subsequent patient care. 

The surgical plan was modified to include taking specimens of aqueous humor and tissue at multiple points during surgery to be analyzed in the laboratory. These specimens included aqueous humor taken prior to entry through the lens capsule, liquefied lens cortex aspirate taken from the cataractous lens in situ, and aqueous humor taken after the cataract was removed but before wound closure. In addition, the anterior capsule was removed in its entirety for in situ hybridization with probe pairs targeting EBOV nucleoprotein genomes. After the cataract was extracted and the intraocular lens implanted, a 10-0 nylon suture was placed as a precautionary measure (Appendix; Video). A conjunctival swab specimen taken after the procedure was sent for EBOV RNA testing in the Emory Serious Communicable Disease Program laboratory onsite using a BioFire FilmArray BioThreat E test (BioFire Defense, https://www.biofiredefense.com). Before machine and tubing breakdown and waste management, RT-PCR was performed on the collected specimens; all results were negative for EBOV. 

**Video:** Cataract surgery in an Ebola virus disease survivor with prior ocular viral persistence.

The extracted lens capsule was formalin-fixed and paraffin-embedded for histopathology. We performed in situ hybridization, with probe pairs targeting genomic EBOV nucleoprotein genes. Anterior capsular thickening was observed, but EBOV RNA was not detected in the anterior capsule tissue (Figure, panels D–F). Transmission electron microscopy of the lens cortex showed vacuolations and examination of thick sections stained with toluidine blue showed fragments of lens cortex; no EBOV particles were observed. Mass spectrometry showed 215 peptides of human origin, with the greatest numbers of peptide spectrum matches observed for beta-crystallin, alpha-crystallin, phakinin, and gamma-crystallin proteins. We observed no EBOV-specific peptide sequences. 

By 1 month after cataract surgery, visual acuity in the patient’s left eye had improved to 20/20, which was maintained at 24-month follow-up. Postoperative retinal examination showed multifocal chorioretinal scars and mild vitreous opacity in the left eye. 

## Conclusions

We report an approach to safe, vision-restoring phacoemulsification surgery for a visually significant cataract in an EVD survivor with previously documented ocular EBOV persistence. This stepwise approach could be used as a model for treatment of EVD survivors from affected areas after an outbreak. Negative results from RT-PCR testing of aqueous humor and liquefied lens cortex specimens provided assurance that no EBOV exposure had occurred during surgery. 

Tropism of ocular tissue and cells of EBOV remains ill-defined in humans. In nonhuman primate survivors of EVD, EBOV RNA has been detected in macrophage reservoirs within the vitreous cavity (*10*). Smith et al. demonstrated, in vitro, that retinal pigment epithelial cells are a potential reservoir for EBOV infection and support viral replication with the release of virus in high titer (*11*); however, they express immunomodulatory molecules linked to ocular immune privilege. 

Because of concerns about viral persistence raised in these studies, our practice had been to ensure that EBOV RNA was not detected before proceeding with ocular surgery. RT-PCR testing conducted 3 months before surgery did not detect EBOV RNA in the patient’s aqueous humor. The Ebola Virus Persistence in Ocular Tissues and Fluids Study in Sierra Leone likewise used a stepwise approach to cataract surgery, requiring a negative result from an aqueous humor aspirate test for EBOV RNA prior to manual small-incision cataract surgery (*12*). This surgery has now been performed successfully for >50 EVD survivors in Sierra Leone and Liberia (*13*), but this approach, in which multiple laboratories were needed for analyses, would not be feasible in many areas affected by EBOV outbreaks. 

Questions remain about the optimal length of time to wait after EBOV infection before performing cataract surgery and about the safety of other types of ophthalmic surgery. Whereas phacoemulsification was performed safely and effectively in this case, it was not done until approximately 27 months after EBOV RNA had been identified in the patient’s aqueous humor. In 2 phases of a previous study, we documented that the aqueous humor tested negative for EBOV RNA at a median of 19 months in one phase and 34 months in the other (*12*). Whether cataract surgery may be performed safely prior to these time points requires better understanding of the kinetics of EBOV entry and clearance from the eye. 

Our team used ultrastructural, genomic, and proteomic assessment on tissues and fluids in a multidisciplinary, multilaboratory approach to mitigate surgical risk. Given the thousands of global EVD survivors with a potential need for eye surgery, a comprehensive understanding of safety precautions for both EVD survivors and their health care providers, as well as of surgical and laboratory approaches for effective eye surgery, will be needed. 

Appendix Additional information on approach to cataract in an Ebola virus disease survivor with prior ocular viral persistence: phacoemulsification surgery, lens biopsy, and laboratory analyses.
