# Correction: Vol. 26, No. 7

**DOI:** 10.3201/eid2609.C12609

**Published:** 2020-09

**Keywords:** errata, erratum, correction

The name of author Xiankun Zeng was misspelled in in Changes in Approach to Cataract Surgery in an Ebola Virus Disease Survivor with Prior Ocular Viral Persistence (J.R. Wells et al.). The article has been corrected online (https://wwwnc.cdc.gov/eid/article/26/7/19-1559_article).

