# Supplementary figures and images for: Approach to Cataract Surgery in an Ebola Virus Disease Survivor with Prior Ocular Viral Persistence
Source: Emerg Infect Dis. 2020 Jul;26(7):1553–6. doi: 10.3201/eid2607.191559 (PMC7323527; doi:10.3201/eid2607.191559)

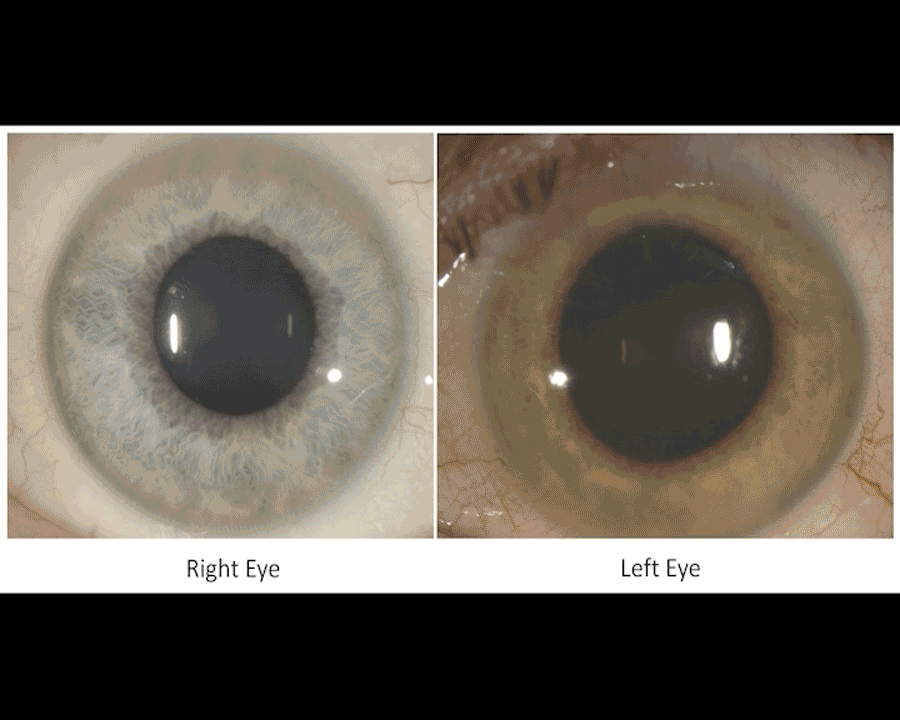

Supplement: Supplementary file 1 [file 19-1559-V.gif]
