# Supplementary material for: Approach to Cataract Surgery in an Ebola Virus Disease Survivor with Prior Ocular Viral Persistence
Source: Emerg Infect Dis. 2020 Jul;26(7):1553–6. doi: 10.3201/eid2607.191559 (PMC7323527; doi:10.3201/eid2607.191559)
Supplement: Appendix — Additional information on approach to cataract in an Ebola virus disease survivor with prior ocular viral persistence: phacoemulsification surgery, lens biopsy, and laboratory analyses. [file 19-1559-Techapp-s1.pdf]

Article DOI: https://doi.org/10.3201/eid2607.191559

Approach to Cataract Surgery in an Ebola Virus Disease Survivor with Prior Ocular Viral Persistence

Appendix

Additional Infection Prevention and Control Precautions

Infection control precautions were vetted with the Emory University Serious Communicable Disease Unit (SCDU), Emory Infection Prevention and Control (IPC), the Anesthesiology service, and the Ophthalmology team before cataract surgery. A risk assessment was performed and, given that the patient's conjunctival surface and aqueous humor had recently tested negative for EBOV RNA by reverse transcription PCR, dilation drops were administered routinely with no specific precautions before surgery. During the cataract surgery, it was not possible to use dedicated eye protection while viewing the field through the microscope, but the microscope eye pieces were thought to offer similar protection to goggles or a face shield. Following the cataract surgery, all equipment and waste remained in the room until confirmation of negative EBOV RNA testing, followed by a terminal room cleaning and waste management precautions guided by Emory SCDU/IPC recommendations.

Detailed Cataract Surgery Procedure

The patient was prepped and draped in usual sterile fashion. A 1-mm side port blade was used to create a inferotemporal paracentesis from which aqueous humor was aspirated with a 27-gauge cannula for EBOV RNA analysis (Video). The surgical team injected 1% preservative-free, intracameral lidocaine, followed by Trypan blue; a dispersive viscoelastic was used to fill the anterior chamber. A 2.4 mm keratome blade was used to create a biplanar clear corneal incision temporally. To reduce the risk of the Argentinian flag sign (i.e., inadvertent tear in the anterior capsule of the lens, often due to pressure from a hypermature cataractous lens), the high-control viscoadaptive Healon5 ophthalmic viscosurgical device (Abbott Medical Optics, http://www.abbottmedicaloptics.com/) was instilled directly over the anterior capsule.

To biopsy the liquefied lens cortex from the cataractous lens for EBOV RNA testing in situ, a 25-gauge needle on a 3-mL syringe was introduced through the anterior capsule, while ensuring central positioning of the needle, and the anterior cortical material was aspirated. An uncomplicated, continuous curvilinear capsulorrhexis was performed. A simcoe cannula was then used to remove additional cortical material for testing. Gentle hydrodissection was performed with balanced saline solution, and the remaining lens material was removed with the phacoemulsification and irrigation-aspiration handpieces. The capsular bag was filled with a cohesive viscoelastic and a single-piece intraocular lens was injected into the capsular bag. The remaining viscoelastic was removed and a final anterior chamber aspirate was obtained for EBOV RNA testing. A 10-0 nylon suture was placed through the main incision as a precautionary measure. A conjunctival swab was performed with a Dacron swab and sent for EBOV RNA testing in the SCDU laboratory onsite by BioFire FilmArray BioThreat-E test (BioFire Defense, https://www.biofiredefense.com).
